# Salivary Biomarkers and Their Application in the Diagnosis and Monitoring of the Most Common Oral Pathologies

**DOI:** 10.3390/ijms21145173

**Published:** 2020-07-21

**Authors:** Lucía Melguizo-Rodríguez, Victor J. Costela-Ruiz, Francisco Javier Manzano-Moreno, Concepción Ruiz, Rebeca Illescas-Montes

**Affiliations:** 1 Biomedical Group (BIO277), Department of Nursing, Faculty of Health Sciences (Ceuta), University of Granada, 51001 Granada, Spain; luciamr@ugr.es; 2 Instituto Investigación Biosanitaria, ibs.Granada, 18012 Granada, Spain; vircoss@ugr.es (V.J.C.-R.); fjmanza@ugr.es (F.J.M.-M.); rebecaim@ugr.es (R.I.-M.); 3 Biomedical Group (BIO277), Department of Nursing, Faculty of Health Sciences, University of Granada, 18016 Granada, Spain; 4 Biomedical Group (BIO277), Department of Stomatology, School of Dentistry, University of Granada, 18071 Granada, Spain; 5 Institute of Neuroscience, University of Granada, 18016 Granada, Spain

**Keywords:** salivary biomarker, cytokines, oral pathology, diagnosis

## Abstract

Saliva is a highly versatile biological fluid that is easy to gather in a non-invasive manner—and the results of its analysis complement clinical and histopathological findings in the diagnosis of multiple diseases. The objective of this review was to offer an update on the contribution of salivary biomarkers to the diagnosis and prognosis of diseases of the oral cavity, including oral lichen planus, periodontitis, Sjögren’s syndrome, oral leukoplakia, peri-implantitis, and medication-related osteonecrosis of the jaw. Salivary biomarkers such as interleukins, growth factors, enzymes, and other biomolecules have proven useful in the diagnosis and follow-up of these diseases, facilitating the early evaluation of malignization risk and the monitoring of disease progression and response to treatment. However, further studies are required to identify new biomarkers and verify their reported role in the diagnosis and/or prognosis of oral diseases.

## 1. Introduction

The gold standard for the identification and diagnosis of oral mucosal diseases is the clinical examination by dental health professionals, followed by histopathological examination of suspicious areas [1,2]. Many diseases of the oral cavity can undergo malignant transformation. Oral squamous cell carcinoma (OSCC) is one of the most frequent oral cancers and still has a five-year survival rate of only 50–65% despite diagnostic and therapeutic advances, in part attributable to diagnostic delay [3]. In most cases of OSCC, the diagnosis is based on the histopathological study of a biopsy. The analysis of saliva, which does not require an invasive procedure, is an attractive alternative option for the diagnosis and prognosis of this oral disease [4,5]. Samples can be readily obtained in a pain-free manner, their processing is relatively simple, their composition is less complex, and they are more stable in comparison to other sources [6,7]. Saliva also offers real-time results, being produced by exocrine glands, and therefore, yielding information on patients at the time the sample is taken [8]. Besides the components secreted by these glands, saliva contains other molecules that can potentially be associated with the disease phenotype and facilitate diagnosis and prognosis, including metabolites, proteins, mRNA, DNA, enzymes, hormones, antibodies, antimicrobial constituents, and growth factors [8,9]. However, it should be noted that some biomarkers detected in saliva are not specific to a particular disease and can be used for the diagnosis of various pathologies. Therefore, it is necessary to consider the different biomarkers that are affected in each disease in order to make a much more specific diagnosis and prognosis.

Salivary biomarkers used to diagnose/monitor diseases include cortisol for Cushing disease or stress disorders [10,11]; C-reactive protein (CRP), creatine kinase isoform MB, and myoglobin for cardiovascular disease [12]; pathogens, nucleic acids, and antibodies for infectious processes [13,14]; α-2-macroglobulin and glycosylated hemoglobin (HbA1c) for diabetes [15]; and various interleukins (ILs), for cancers, gut diseases, and muscle or joint disorders [16]. Therefore, the objective of this review was to determine the potential usefulness of different salivary biomarkers to assist the diagnosis and prognosis of oral cavity diseases. 

## 2. Biomarkers in Saliva in Different Oral Diseases

Among the numerous diseases of the oral cavity, this review focuses on the following: oral lichen planus (OLP), periodontitis (PD), and primary Sjögren’s syndrome (pSS) for their high prevalence; oral leukoplakia for its malignant transformation potential, also shared by OLP; peri-implantitis for its possible negative effects on the medium- and long-term success of dental implantation; and medication-related osteonecrosis of the jaw (MRONJ) for its potential impact on the oral and general quality of life of patients. Salivary biomarkers can be useful for the diagnosis, monitoring, and even prognosis of all of these diseases (Table 1).

### 2.1. Oral Lichen Planus

OLP is a chronic inflammatory disease that affects the oral mucosa, including the tongue and gingival tissues. OLP is estimated to affect 1.01% of the population worldwide, with a higher rate in Europe (1.43%) [109]. It is considered a potentially malignant disease and with a 1.14% probability of oral cancer development [110]. Although no consensus has been established on its etiopathogenesis, the onset and progression of OLP have been attributed to an immunological mechanism responsible for cutaneous manifestations such as erythema, white streaks, papillae, or ulcerations [111]. Abnormal activation of the immune system is mediated by the signaling of different molecules that have been investigated as possible salivary biomarkers of OLP. 

Cortisol is the main glucocorticoid that regulates processes and behaviors, including immunoregulation. High cortisol levels are attributed to the presence of stress and may trigger immunological disorders [112]. Salivary cortisol values have been widely studied in relation to the effects of stress- and anxiety-related psychological factors on immune diseases. Elevated salivary cortisol values have been observed in patients diagnosed with OLP than in those without this disease [17,18,19], and it has been suggested that there is a link between high cortisol levels and psychological strains as triggering factors of OLP [20]. Given reports that stress can be responsible for the recurrence of OLP, cortisol has been proposed as a possible diagnostic marker for this disease [21]. 

The role of oxidative stress in OLP has been investigated [113,114], and the pathogenesis of this disease has been related to nitric oxide (NO) and reactive oxygen species (ROS) [22]. Numerous studies have described higher salivary NO levels in patients with OLP than in healthy individuals [19], and elevated levels have been associated with a more severe disease progression through the production of mucosal lesions [22,23]. For their part, ROS have been associated with cellular oxidative stress, but there is no clear consensus on the relationship between their salivary concentrations and oxidative damage in the tissues of patients with OLP [22,24]. CRP is frequently used as a marker of inflammation, and its salivary levels are higher in patients with OLP than in healthy individuals [22,25,26], indicating a potential role in monitoring the progression of this disease [26].

Among cytokines, salivary levels of tumor necrosis factor α (TNF-α), IL1, IL4, IL6, and IL8 have been described as relevant biomarkers for OLP diagnosis and prognosis [19]. TNF-α has been studied in relation to OLP since the last century [115]. This pro-inflammatory and immunomodulatory cytokine stimulates the acute phase of inflammation, leading to the synthesis of other pro-inflammatory cytokines (e.g., IL1 and IL6) and the activation of T and B cells. It is therefore considered to mediate autoimmune and inflammatory processes, including OLP. Elevated salivary TNF-α levels have been observed in patients with OLP [19,31,32,33], and TNF-α is found to act at the onset of OLP and during its progression [31]. IL1α and IL1β both stimulate various cell populations, including T-helper (Th) lymphocytes, by increasing IL2 secretion and IL2 receptor (IL-2R) expression. They can stimulate their own production and that of other cytokines such as IL6 and IL8, playing a major role in mediating inflammation and regulating the immune response [57,58]. Higher levels of both IL1α and IL1β have been recorded in patients with OLP than in healthy individuals [19,32]. 

IL4 is a cytokine produced mainly by Th2 lymphocytes that exerts anti-inflammatory action by blocking the synthesis of pro-inflammatory cytokines. Although elevated salivary IL4 levels been found in OLP patients [19,61], there is insufficient evidence to support its usefulness as a biomarker of disease progression [32,62]. IL6 is a pleiotropic cytokine secreted by different cells and is considered to be pro-inflammatory and to mediate immune and inflammatory responses [116]. Significantly higher salivary IL6 levels have been found in OLP patients [19,32,64,65], attributed to the overexpression of tripartite motif-containing 21 (TRIM21), which participates in the regulation of intracellular and immune processes [117]. The association of IL6 with the onset of lesions and with advanced stages of OLP has led to its proposal as a biomarker of the response to treatment [32,66]. Another pro-inflammatory cytokine, IL8, is generated in response to damage, and its salivary levels are higher in patients with OLP than in healthy individuals [19,76,77]. Salivary levels of IL6 and IL8 have been related to the severity of OLP [32], although some authors have described salivary IL8 as a more reliable OLP biomarker [66,78].

### 2.2. Periodontitis

A wide variety of etiological factors have been implicated in PD, a severe gingival infection that can lead to the destruction of periodontal ligament and alveolar bone [118,119]. Most cases have a bacterial etiology, generating an anti-inflammatory response mediated by cytokines, chemokines, and other biomolecules [120,121,122]. IL1β, TNF-α, IL6, and the receptor activator of nuclear factor κB ligand (RANKL), among other cytokines, are known to be involved in immune response regulation in PD and to play a key role in its development [36,37]. 

IL1α is produced by cells in numerous periodontal tissues and plays an important role in the immune response to plaque bacteria in PD and other oral diseases. This cytokine frequently acts synergistically with TNF-α and prostaglandin E2 (PGE2) to produce various vascular inflammation-related modifications, and this action is especially important in the migration of neutrophils from the bloodstream to the periodontium. The increased expression of IL1β, TNF-α, and PGE2 in oral cavity fluids and tissues in PD suggests their potential use as biomarkers of its presence and progression. These proteins participate in the activation of osteoclasts, the secretion of infiltrating neutrophils, and the resorption of alveolar bone in chronic PD [51,52]. The presence of IL1β in saliva has enabled discrimination of individuals with PD from those without this disease [35,38,39,46,47,48,49,50], and its levels have been correlated with alveolar bone loss levels [53].

Salivary TNF-α levels are very low and frequently undetectable and, therefore, are of little prognostic or diagnostic value [38,39]. In addition, findings have been controversial, with reports of significantly elevated [34] and significantly reduced [35] salivary TNF-α levels in patients with PD. 

Another frequently analyzed cytokine in oral cavity disease is IL6. It is produced by numerous cells of the periodontium in response to IL1β and TNF-α secretion, playing a major role in the activity of immune cells and osteoclasts and the inflammatory response to bacterial plaque formation [37,69,70]. Elevated salivary IL6 expression was observed in patients with PD by some authors [38,63,67] but not by others [27,39,49,68]. Among other salivary cytokines studied in this context, elevated IL4 levels and reduced IL17 levels [63] have been reported in patients with PD. Salivary levels of monocyte chemoattractant protein 1 have also been associated with this disease [85,86].

Salivary levels of RANKL, osteoprotegerin (OPG), and osteocalcin (OSC) have also been studied in patients with PD, mainly to explore the relationship of these biomarkers with bone loss. However, the results have been contradictory, with this association being reported by some authors [83] but not by others [34,84].

Many other salivary biomarkers have been studied in relation to PD, including inflammatory markers, cell activity markers, and growth factors. Inflammatory markers found to be elevated in PD patients include CRP and calprotectin, a known marker of inflammatory bowel disease, and frequently analyzed in feces [27,28,29,30]. Among cell activity markers, elevated levels of alkaline phosphatase (ALP), lactate dehydrogenase (LDH), aspartate aminotransferase (AST), alanine aminotransferase (ALT), and matrix metalloproteases (MMPs) have been associated with PD [67,87,88,89,90,91,92]. MMP-8 has been described as a more useful salivary biomarker of PD in comparison to IL1β [27,39,47,48,67,87,93]. In addition, elevated MMP-9 levels [27,35] but reduced tissue inhibitor metalloproteinase-1 (TIMP-1) levels have been detected in the saliva of patients with PD [93,97]. Available data on the association of salivary growth factors with PD are limited and imprecise. On the other hand, hepatocyte growth factor (HGF) has been related to PD as a possible mediator of apical epithelial migration [98,99,100]. 

Liukkonen studied 220 patients classified by PD type and reported that salivary levels of IL-17A and IL23 were higher in patients with localized periodontitis, whereas IL1β levels were higher in those with generalized periodontitis [54]. Lira Junior et al. observed higher salivary MMP-8 levels in patients with aggressive PD than in healthy individuals [94]. Isaza Guzmán et al. described salivary levels of nod-like receptor family pyrin domain-containing protein 3 (NLRP3) and IL1β as indicators of the presence and severity of chronic or aggressive PD that may be useful for preventive and/or therapeutic purposes [55]. 

These types of salivary biomarkers may be useful for the diagnosis, follow-up, and even prognosis of PD and support the delivery of optimal care.

### 2.3. Primary Sjögren’s Syndrome

pSS is a chronic systemic autoimmune disease that damages salivary and lacrimal glands [123]. It is the second most frequent autoimmune rheumatic disease, with a prevalence of around 1% [124,125]. The immunopathogenic mechanism is based on the pathological hyperactivity of B lymphocytes, expressed as a T lymphocyte-mediated increase in antibody production and the activation of interferon production pathways [126]. The glandular destruction can often generate a chronic inflammatory response in the salivary glands that result in xerostomia [127], associated with multiple oral complications such as oral candidiasis, caries, and PD. Early diagnosis of SS is essential to avoid these adverse effects, and clinicians should be alert to its signs and symptoms in their patients. 

The diagnosis of pSS is generally based on a series of clinical and histopathological signs and symptoms that are often difficult to interpret, and different classifications have been published [128,129,130]. This has prompted research into the diagnostic and prognostic value of salivary biomarkers for pSS to facilitate early treatment and reduce the associated complications. In comparison to healthy patients, patients with pSS have an increased salivary expression of S100A proteins, directly related to IL-12 production pathways [81]; proteins vital for innate MHC class I cellular regulation (NGAL) and T-cell activation (CD44) [101]; and β-2 macroglobulin (B2M), which has been significantly correlated with lymphocyte infiltration in labial salivary glands [102]. Recently, auto-antibodies to salivary protein-1 (SP-1), parotid secretory protein (PSP), and carbonic anhydrase VI (CA-6) [103,104] have been proposed as biomarkers for an early pSS diagnosis to reduce complications and improve the prognosis. Further studies are warranted to identify salivary biomarkers for the prognosis of patients with pSS and for evaluating their progression and response to treatments.

### 2.4. Oral Leukoplakia

Oral leukoplakia is characterized by a whitish plaque in oral mucosa that cannot be removed by scraping, and it predisposes patients to oral cancer development [131]. It is closely related to the consumption of tobacco [132] and has also been associated with alcohol use [133], with fungal [134], bacterial [135,136], and viral [137,138] infections, and with hormonal disorders [139,140]. It is one of the most common premalignant lesions of the oral cavity, being responsible for around 11% of squamous cell carcinomas [141] and 3.5% of malignant transformations with a range between 0.13% and 34% [142]. The risk of malignant transformation is evaluated by taking a biopsy for the analysis of histopathological markers, including signs of dysplasia such as asymmetrical epithelial stratification, pleomorphism, myoepithelial basocellular hyperplasia, hyperchromatic nuclei, and dyskeratosis [143]. There is increasing interest in less invasive diagnostic procedures, including the analysis of pro-inflammatory cytokines.

Deepthi et al. reported that TNF-α acts as a prognostic marker of OSCC, observing elevated salivary TNF-α levels in patients with dysplasia and suggesting that this cytokine may be useful to monitor the malignant transformation of oral leukoplakia [40]. Other authors proposed that TNF-α can serve as a biomarker for the early diagnosis of pre-oral cancer, given that the levels of this cytokine and various ILs are higher in patients with more advanced precancerous lesions [41]. TNFα polymorphisms have also been associated with precancerous oral lesions [42]. Numerous authors have explored the use of ILs as salivary markers for the diagnosis and prognosis of oral leukoplakia. It is generally reported that IL6 and IL8 levels are elevated in patients with oral leukoplakia in comparison to healthy individuals [41,71,72,73]. These angiogenic mediators are suggested as potential salivary biomarkers for early cancer detection, and they are associated with tumor growth and increased blood vessel density [74]. Other ILs investigated in relation to this disease include IL37, found to be elevated in patients with oral leukoplakia [82], and IL10, whose levels have not been significantly associated with premalignant oral lesions [42,80]. However, Brailo et al. observed no difference in salivary TNF-α levels between healthy individuals and patients with oral leukoplakia or oral cancer [43]. Wenghoefer et al. also found no positive relationship between the inflammation markers IL1 β, IL6, IL8, IL10, TNF-α, or COX2 and the development of these oral lesions, even observing a lower expression of IL1β and IL10 in patients with these diseases than in healthy individuals [44]. 

Besides cytokines, it has been reported that salivary levels of the enzyme LDH, whose expression is closely related to cell necrosis, are elevated in patients with oral leukoplakia and even higher in those with OSCC. Therefore, this marker may be useful to evaluate the risk of malignant transformation of oral leukoplakia [105,106]. Endothelins and growth factors such as transforming growth factor β (TGFβ) and epidermal growth factor (EGF) have also been investigated in relation to oral leukoplasia. However, no significant relationship has been found between their salivary levels and the diagnosis or prognosis [80,107,108].

Data on the usefulness of salivary biomarkers in oral leukoplakia are not conclusive, and further research is warranted to verify the results obtained and to explore new candidate biomolecules for this purpose.

### 2.5. Peri-Implantitis

Peri-implantitis is an inflammatory disease that destroys hard and soft tissues around dental implants and is one of the main causes of medium- and long-term implant failure. It is triggered by the accumulation of bacteria on the implant surface, generating mucosal inflammation [144]. It is a progressive and irreversible peri-implant disease accompanied by bone resorption, reduced osseointegration, the formation of peri-implant pockets, and purulent secretions [145,146,147]. 

The most widely studied biomarkers of this disease include pro-inflammatory cytokines IL1β, IL6, IL12, IL17, and TNF-α; anti-inflammatory cytokines IL4 and IL10; osteoclastogenic cytokines RANK, RANKL, and OPG; antioxidant proteins (e.g., urate, malondialdehyde, ascorbate, and myeloperoxidase); and the chemokine IL8 [148]. Peri-implantitis has been associated with increased salivary levels of IL1β, [45,56] IL6, and IL10 levels [45,75], and these interleukins have been proposed as potentially useful markers for the early diagnosis and follow-up of this disease [45,75]. Salivary IL8 and IL12 levels were found to be higher in patients with peri-implantitis than in those with peri-implant mucositis [79]. TNF-α levels were also reported to be higher in patients with peri-implantitis than in healthy individuals [45].

Peri-implantitis is a cause of medium- and long-term implant failure, and the identification of biomarkers of this disease would support the implementation of appropriate preventive and therapeutic measures.

### 2.6. Medication-Related Osteonecrosis of the Jaw

MRONJ is a severe drug-related complication associated with the use of antiresorptive medication (e.g., bisphosphonates [BPs] and RANKL inhibitors) and with anti-angiogenic medication [149]. BP-related osteonecrosis of the jaw was first described by Robert Marx in 2003 [150]. After the implication of other drugs in maxillary osteonecrosis, such as RANKL inhibitors (denosumab) or VEGF-inhibiting anti-angiogenic drugs, the American Association of Oral and Maxillofacial Surgeons changed the term “BRONJ” to “MRONJ” [151]. MRONJ has been associated with various possible etiologies, including reduced bone turnover and the consequent accumulation of microfractures, avascular necrosis due to anti-angiogenic effects, impaired viability of fibroblasts, and oral keratinocytes; and osteoblast physiology disorders [152,153,154,155].

Difficulties in the early diagnosis of MRONJ, which relies exclusively on clinical findings, has led a small number of researchers to study candidate biomarkers for this purpose [156,157]. Yatsuoka et al. found significantly increased salivary levels of hypotaurine in patients with early-stage MRONJ in comparison to healthy individuals [158]. Hypotaurine is an intermediate in the biosynthesis of taurine, which acts as an antioxidant in cellular defense against oxidative stress, and the detection of increased salivary levels may assist the early diagnosis of MRONJ. Bagan et al. observed a significant increase in the levels of IL1ɑ, IL1β, interleukin-1 receptor antagonist (IL-1RA), and IL6 in the saliva of patients with MRONJ in comparison to healthy individuals [59,60]. These ILs are closely related to the inflammatory process and alveolar bone loss produced in MRONJ, and their analysis may, therefore, be useful in the detection of this disease. Thumbigere-Math et al. described elevated salivary levels of MMP-9 in patients with MRONJ, proposing this protein as a biomarker of this disease [95,96].

Measurement of systemic parameters in MRONJ monitoring represents a long-lasting and ongoing debate with no clear results until now. Different bone biomarkers have been proposed for the risk prevention of MRONJ like OSC, C-terminal telopeptide of collagen I, N-terminal telopeptides, ALP, and parathyroid hormone [159,160,161]. However, there is insufficient evidence that these biomarkers are effective in predicting the diagnosis and prognosis of MRONJ. The availability of reliable salivary biomarkers for the early diagnosis of MRONJ could make a major contribution to the correct management of these patients, reducing their morbidity.

## 3. Conclusions

In conclusion, salivary levels of various biomarkers are known to change in the presence of oral cavity diseases and can, therefore, be useful for their diagnosis and prognosis. Some biomarkers, such as pro-inflammatory cytokines, are common to many of these diseases, whereas others are more specific. Their evaluation in saliva offers clinicians a valuable non-invasive procedure as a complement to clinical findings, and further research is warranted to establish reliable salivary biomarkers for different diseases of the oral cavity.

## Figures and Tables

**Table 1:** Salivary Biomarkers involved in main oral pathologies diagnosis and prognosis.

Biomarker		Oral Pathology	Salivary Levels in Diagnosed Patients	Clinical Relevance
Cortisol		OLP	Increased levels [17,18,19]	Diagnosis and recurrence of the pathology [20,21]
Nitric Oxide		OLP	Increased levels [19]	Prognosis and presence of ulcers [22,23]
ROS		OLP	Unaltered levels [22]	Cellular oxidative stress [22,24]
CRP		OLP	Increased levels [22,25,26]	OLP progression [26]
		PD	Increased levels [27,28,29,30]	PD prognosis (modulation of the inflammation) [27,28,29,30]
TNF- α		OLP	Increased levels [19,31,32,33]	OLP diagnosis, commencement and progression [19,31]
		PD	Increased levels [34] Decreased levels [35]	Uncertain diagnosis, and prognosis role [36,37,38,39]
		OL	Increased levels [40,41,42] Unaltered levels [43,44]	OL prognosis (malignant transformation, pre-oral cancer, and precancerous marker) [40,41,42]
		PI	Increased levels [45]	Diagnosis of the pathology [45]
IL1	IL1β	PD	Increased levels [35,38,39,46,47,48,49,50]	Diagnosis and progression (inflammatory modulation, severity-bone resorption, generalized PD and PD severity) [51,52,53,54,55]
		OL	Unaltered levels [44]	-
		PI	Increased levels [45,56]	Diagnosis of the pathology [45,56]
	IL1α & IL1β	OLP	Increased levels [19,32]	Immune and inflammatory response modulator [57,58]
		MRONJ	Increased levels [59,60]	MRONJ diagnosis [59,60]
IL1RA		MRONJ	Increased levels [59,60]	MRONJ diagnosis [59,60]
IL4		OLP	Increased levels [19,61]	IL4 is not a good salivary marker for OLP prognosis [32,62]
		PD	Increased levels [63]	-
IL6		OLP	Increased levels [19,32,64,65]	OLP prognosis (severity and wound marker). IL6 salivary marker is a good option for monitoring the treatment response [32,66]
		PD	Increased levels [38,63,67] Unaltered levels [27,39,49,68]	PD prognosis (inflammatory modulator) [37,69,70]
		OL	Increased levels [41,71,72,73] Unaltered levels [44]	OL prognosis (tumor growth and higher blood vessel density) [74]
		PI	Increased levels [45,75]	Early diagnosis and prognostic value [45,75]
		MRONJ	Increased levels [59,60]	MRONJ diagnosis [59,60]
IL8		OLP	Increased Levels [19,76,77]	IL8 is a solid salivary biomarker for OLP severity [32,66,78]
		OL	Increased levels [41,71,72,73] Unaltered levels [44]	OL prognosis (tumor growth and higher blood vessel density) [74]
		PI	Increased Levels [79]	PI diagnosis [79]
IL10		OL	Increased Levels [42,80] Unaltered levels [44]	Uncertain association with premalignant oral lesions [42,80]
		PI	Increased levels [45,75]	Early diagnosis and prognostic value [45,75]
IL12		pSS	Increased Levels [81]	Diagnostic and prognostic value [81]
IL17		PD	Increased levels [54,63]	Localized periodontitis [54]
IL23		PD	Increased levels [54]	Localized periodontitis [54]
IL37		OL	Increased Levels [82]	
RANKL		PD	Increased levels [83] Unaltered levels [34,84]	Uncertain prognosis value (bone loss) [34,83,84]
MIP-1		PD	Increased levels [85,86]	Diagnosis [85,86]
OPG		PD	Decreased levels [83] Unaltered levels [34,84]	Uncertain prognosis value (bone loss) [34,83,84]
OSC		PD	Decreased levels [83] Unaltered levels [34,84]	Uncertain prognosis value (bone loss) [34,83,84]
ALP		PD	Increased levels [67,87,88,89,90,91,92]	Diagnosis of the pathology [67,87,88,89,90,91,92]
LDH		PD	Increased levels [67,87,88,89,90,91,92]	Diagnosis of the pathology [67,87,88,89,90,91,92]
AST		PD	Increased levels [67,87,88,89,90,91,92]	Diagnosis of the pathology [67,87,88,89,90,91,92]
ALT		PD	Increased levels [67,87,88,89,90,91,92]	Diagnosis of the pathology [67,87,88,89,90,91,92]
MMP8		PD	Increased levels [27,39,47,48,67,87,93]	Very useful salivary biomarker for the diagnosis of PD [27,39,47,48,67,87,93] and PD severity [94]
MMP9		PD	Increased levels [27,35]	Diagnosis [27,35]
		MRONJ	Increased levels [95,96]	MRONJ diagnosis [95,96]
TIMP1		PD	Decreased levels [93,97]	PD prognosis (advanced PD) [93]
HGF		PD	Increased levels [98,99]	Prognosis of the pathology [98,99,100]
NLRP3		PD	Increased levels [55]	PD severity and chronicity. Also useful as a salivary biomarker for preventive or therapeutic purposes [55]
CD44		pSS	Increased levels [101]	Diagnostic and prognostic value [101]
B2M		pSS	Increased levels [102]	Diagnostic and prognostic value [102]
SP1		pSS	Increased levels [103,104]	Early diagnosis and prognostic value [103,104]
PSP		pSS	Increased levels [103,104]	Early diagnosis and prognostic value [103,104]
CA6		pSS	Increased levels [103,104]	Early diagnosis and prognostic value [103,104]
LDH		OL	Increased levels [105,106]	Risk of malignant transformation of OL [105,106]
TGFβ		OL	Unaltered levels [80,107,108]	Uncertain diagnosis and prognosis value [80,107,108]
EGF		OL	Unaltered levels [80,107,108]	Uncertain diagnosis and prognosis value [80,107,108]

OLP: Oral Lichen Planus; PD: Periodontitis; pSS: Primary Sjögren Syndrome; OL: Oral Leukoplakia; PI: Periimplantitis; MRONJ: Medication-Related Osteonecrosis of the Jaw.

## Abbreviations

OSCC	oral squamous cell carcinoma
ILs	interleukins
CRP	C-reactive protein
OLP	oral lichen planus
PD	periodontitis
pSS	primary Sjögren’s syndrome
MRONJ	medication-related osteonecrosis of the jaw
NO	nitric oxide
ROS	reactive oxygen species
TNF-α	tumor necrosis factor α
Th	T-helper
IL-2R	IL-2 receptor
TRIM21	tripartite motif-containing 21
RANKL	receptor activator of nuclear factor κB ligand
PGE2	prostaglandin E2
OPG	osteoprotegerin
OSC	osteocalcin
ALP	alkaline phosphatase
LDH	lactate dehydrogenase
AST	aspartate aminotransferase
ALT	alanine aminotransferase
MMPs	matrix metalloproteases
TIMP-1	tissue inhibitor metalloproteinase-1
HGF	hepatocyte growth factor
NLRP3	nod-like receptor family pyrin domain containing protein 3
SP-1	salivary protein-1
PSP	parotid secretory protein
CA-6	carbonic anhydrase VI
TGFβ	transforming growth factor β
EGF	epidermal growth factor
BPs	bisphosphonates
IL-1RA	interleukin-1 receptor antagonist
